# Reference database of total retinal vessel surface area derived from volume-rendered optical coherence tomography angiography

**DOI:** 10.1038/s41598-022-07439-2

**Published:** 2022-03-07

**Authors:** Peter M. Maloca, Silvia Feu-Basilio, Julia Schottenhamml, Philippe Valmaggia, Hendrik P. N. Scholl, Josep Rosinés-Fonoll, Sara Marin-Martinez, Nadja Inglin, Michael Reich, Clemens Lange, Catherine Egan, Sandrine Zweifel, Adnan Tufail, Richard F. Spaide, Javier Zarranz-Ventura

**Affiliations:** 1 Institute of Molecular and Clinical Ophthalmology Basel, 4031 Basel, Switzerland; 2 Department of Ophthalmology, University Hospital Basel, 4031 Basel, Switzerland; 3 Institut Clínic d’Oftalmologia, Hospital Clínic de Barcelona, 08036 Barcelona, Spain; 4 Pattern Recognition Lab, University Erlangen-Nürnberg, 91058 Erlangen, Germany; 5 Eye Center, Medical Center - University of Freiburg, Faculty of Medicine, University of Freiburg, Freiburg, Germany; 6 Moorfields Eye Hospital NHS Foundation Trust, London, EC1V 2PD UK; 7 University Hospital Zurich, Frauenklinikstrasse 24, 8091 Zurich, Switzerland; 8 University of Zurich, Rämistrasse 71, 8006 Zürich, Switzerland; 9 Vitreous-Retina-Macula Consultants of New York, New York, NY USA; 10 Institut de Investigacions Biomediques August Pi i Sunyer (IDIBAPS), 08036 Barcelona, Spain

**Keywords:** Health care, Medical imaging

## Abstract

Optical coherence tomography angiography (OCTA) enables three-dimensional, high-resolution, depth-resolved flow to be distinguished from non-vessel tissue signals in the retina. Thus, it enables the quantification of the 3D surface area of the retinal vessel signal. Despite the widespread use of OCTA, no representative spatially rendered reference vessel surface area data are published. In this study, the OCTA vessel surface areas in 203 eyes of 107 healthy participants were measured in the 3D domain. A Generalized Linear Model (GLM) model analysis was performed to investigate the effects of sex, age, spherical equivalent, axial length, and visual acuity on the OCTA vessel surface area. The mean overall vessel surface area was 54.53 mm^2^ (range from 27.03 to 88.7 mm^2^). OCTA vessel surface area was slightly negatively correlated with age. However, the GLM model analysis identified axial length as having the strongest effect on OCTA vessel surface area. No significant correlations were found for sex or between left and right eyes. This is the first study to characterize three-dimensional vascular parameters in a population based on OCTA with respect to the vessel surface area.

## Introduction

For decades, fluorescein angiography (FA) has been the gold standard for retinal vessel imaging^1^ despite the fact that it is an invasive^2,3^ and time-consuming procedure that causes mild to severe adverse reactions. In addition, fluorescein angiography does not show the radial peripapillary capillary network, the intermediate capillary plexus or the deep capillary plexus^4^. Thus, the fact of the matter is FA does not adequately image retinal blood flow.

In this context, optical coherence tomography angiography (OCTA) has been successfully introduced as a depth resolved imaging technique that displays the movement of blood within the vessels with the help of intrinsic signals^4–6^ and without the need for a dye injection^7^. Consequently, OCTA has evolved into a fast, safe, and frequently used ophthalmic imaging technology^8^ that demonstrates great promise in terms of improving our understanding of the physiology and pathophysiology of the eye^6^.

For example, in diabetic retinopathy, OCTA provided capillary perfusion density maps for the quantification of the deep capillary layers, which aided in documenting disease progression and in assessing risk stratification^9–11^. Furthermore, OCT and OCTA were applied in the diagnosis, monitoring, and indications for therapeutic interventions in age-related macular degeneration^12,13^, which might affect more than 20% of the aging population^14^. In addition to magnetic resonance imaging, OCT and OCTA have emerged as beneficial imaging tools in multiple sclerosis^15^. The retinal vessel volume-rendering showed the altered microcirculation in the macula, for example in fovea plana, which is important because these eyes cannot fit into the scheme of normative data banks offered by the commercial manufacturers^16^.

Although the information obtained from OCTA is three dimensional, a compromise between the amount of information and the display capabilities^17–20^ was made by condensing the extracted information to a one-pixel thick planar view of selected layers which is currently used as the standard display method^21,22^. Although this trade-off seems reasonable, blood vessels are three dimensional structures designed to carry blood and facilitate exchange so that their three dimensional course^23^ is incompletely captured by the ordinary planar en face views.

As a further limitation to this simplified visualization of OCTA data, it can be stated that previous OCTA investigations are based in part of a relatively small number of subjects^19,24^ and that controversial values for age^19,25,26^ and sex^19,27^ and correlations for axial length (AL)^28,29^ were reported.

A more appropriate way to evaluate OCTA features is in three dimensions to assess the physical characteristics of the vessels including such as the retinal vessel surface area which is particularly important for the metabolic exchange in the retina.

Therefore, utilizing the representation of three-dimensional OCTA data allows us in this study to expand the scope of the current 2D OCTA image display method by means of describing a fully automatic technology for the 3D rendering of OCTA in healthy eyes, providing a 3D rendered normative raw database of the naturally occurring variations in the retinal vascular surface area in healthy individuals, and determining the association of sex, age, spherical equivalent (SE), AL, and visual acuity (VA) with OCTA retinal vascular surface area.

## Results

### Summary statistics and data plots

The OCTA measurements from 203 eyes (102 left, 101 right) of 107 subjects (61.7% female) were included. Five out of 203 eyes were pseudophakic, and 97.5% of the eyes were phakic. Twenty-three eyes were excluded because of the presence of coronary heart disease (n = 2), peripheral vascular disease (n = 10), high myopia (n = 2), amblyopia (n = 6), or cataract (n = 1).

**Figure 1:**
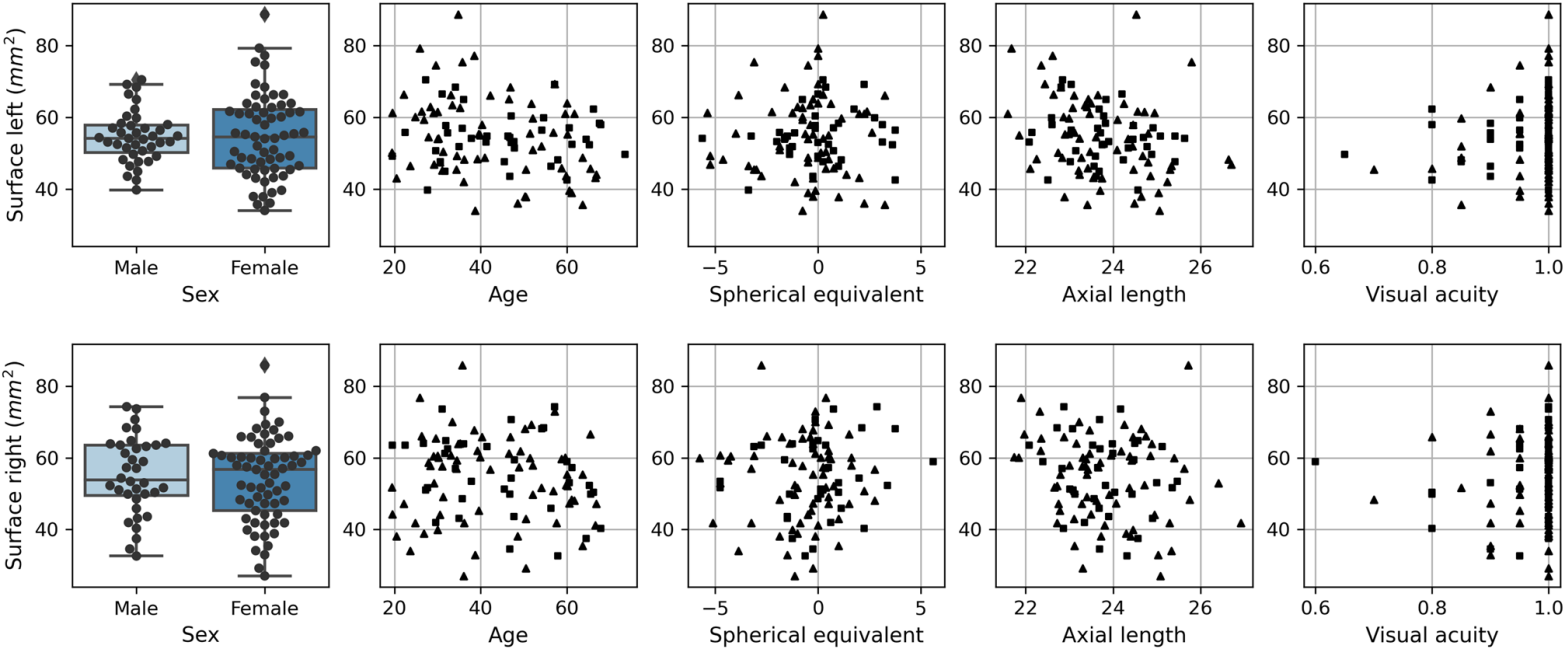
Univariate box plots and scatter plots. Plots in the first row are based on data from the left eyes (OS). Plots in the second row are based on data from the right eyes (OD). Boxplots are grouped by sex. Scatter plots are drawn for age, spherical equivalent, axial length, and visual acuity. Males (females) are indicated by squares (triangles) in the scatter plots. Optical coherence tomography angiography retinal vessel surface area measurements are in mm^2^.

Overall mean OCTA vessel surface was 54.53 mm^2^ (range from 27.03 to 88.70 mm^2^). To avoid correlation effects between the eyes, further results are splitted by right and left eyes. Mean surface areas in males were 55.00 mm^2^ (range from 32.59 to 74.27 mm^2^), and 54.52 mm^2^ (range from 39.74 to 70.48 mm^2^), for the right and left eye, respectively. Mean surface areas in females were 54.07 mm^2^ (range 27.03 to 85.83 mm^2^) and 54.75 mm^2^ (range from 33.98 to 88.70 mm^2^) for right and left eyes, respectively. For the participants, the width of range from minimum to maximum varies up to 56% for males and up to 38% for females. The summary statistics are presented in Table 1, and the data are plotted in Fig. 1. All the measured raw data are summarized in Supplementary raw data S1 (right eyes) and Supplementary adjusted raw data S2 (left eyes). Also included are the proposed corrections for Ocular Magnification Effects (OME).

**Table 1:** Summary statistics of age, spherical equivalent (SE), axial length (AL), visual acuity (VA), and optical coherence tomography angiography (OCTA) retinal vessel surface area measurements.

	Age	SE	AL	VA	Surface overall	Surface male left	Surface female left	Surface male right	Surface female right
Count	203	197	200	202	203	39	63	36	65
Mean	43.39	− 0.41	23.8	0.97	54.53	54.52	54.75	55	54.07
S.d.	14.05	2.04	1.03	0.07	10.8	7.2	11.66	10.79	11.89
Min	19.37	− 5.75	21.59	0.6	27.03	39.74	33.98	32.59	27.03
25%	31.08	− 1.38	23.07	0.95	47.25	50.14	45.76	49.54	45.26
50%	41.25	− 0.25	23.68	1	54.56	54.13	54.56	53.88	56.72
75%	56.84	0.5	24.49	1	61.6	57.83	62.2	63.56	61.21
Max	73.49	5.59	26.92	1	88.7	70.48	88.7	74.27	85.83

Summary statistics are calculated from 203 eyes (102 left and 101 right). Count indicates the number of eyes for which the respective measurements were available. Mean, s.d., min, 25%, 50%, 75%, and max indicate mean, standard deviation, minimum, 1st quartile, 2nd quartile, 3rd quartile, and maximum, respectively. Surface measurements are in mm^2^.

Similar but not significant results were found in both sexes and are summarized in Fig. 1 and Table 1. In the left eye, the standard deviation was higher in females than in males.

### Correlation analysis

Pearson correlation coefficients between OCTA retinal vessel surface area and age were − 0.11 and − 0.21 for right and left eyes, respectively. Testing for non-correlation revealed no significant correlation in right eyes (*p*-value: 0.282) but a significant negative correlation in left eyes (*p*-value: 0.033). Pearson correlation coefficients between OCTA retinal vessel surface area and AL were − 0.20 and − 0.26 with *p*-values 0.049 and 0.009 in right and left eyes, respectively.

### Generalized linear model (GLM) analysis

A GLM analysis was performed to investigate the effects of independent variables, such as age, sex, SE, AL, and VA, on OCTA vessel surface area. The analysis (Table 2) showed that, in the left and right eyes, the main effect of AL had a significant effect on the vessel surface. Regarding AL, the effect was clearer in the left eye (p < 0.001) than in the right eye (p = 4.9e−2). In the right eye, a two-way interaction, sex:SE, was found to have a significant effect at a significance level of 0.05. Other main effects and two-way interactions were not significant in either eye at a significance level of 0.05.

**Table 2:** Generalized linear model (GLM) analysis results.

Effect	Right eyes			Left eyes		
	F value	Pr (> F)	Sign	F value	Pr (> F)	Sign
Age	0.5449	0.46256		2.2139	0.14075	
Sex	0.3855	0.5364		2.7446	0.10155	
SE	0.0007	0.97953		2.6764	0.10583	
AL	3.979	0.04944	*	23.6357	5.81E-06	***
VA	0.6933	0.40748		3.9005	0.05177	
Age:sex	2.3644	0.12803		2.0895	0.15227	
Age:SE	0.2595	0.61184		0.3691	0.54522	
Age:AL	0.3242	0.57067		0.1925	0.66204	
Age:VA	0.3794	0.53968		0.1254	0.72424	
Sex:SE	4.3716	0.03968	*	1.6124	0.20788	
Sex:AL	1.3961	0.24084		2.4731	0.1198	
Sex:VA	0.3656	0.5471		0.0375	0.84688	
SE:AL	0.5191	0.47328		0.0001	0.9924	
SE:VA	0.2368	0.62783		0.0222	0.88192	
AL:VA	0.9001	0.34558		0.315	0.57619	

A GLM analysis was performed for the left and right eyes separately. The main effects of the independent variables, age, sex, spherical equivalent (SE), axial length (AL), and visual acuity (VA), and all the possible two-way interactions were taken into consideration. p-values were calculated using the *F*-test statistic. The “Sign” columns indicate significance codes: 0, '***'; 0.001, '**'; 0.01, '*'; 0.05 '.'; and 0.1, ' ' 1.

### Right vs. left eyes

A paired two-sided *t*-test on the OCTA vessel surface area performed to compare the left and right eyes yielded a p-value of 0.76 (value of *t*-test statistic, 0.306). There was no significant difference in the OCTA retinal surface area between the left and right eyes.

### Age, SE, and VA

In the age group analysis, no impact on vessel surface area was found. Similarly, no significant effects of SE or VA were observed.

## Discussion

Optical coherence tomography angiography has been rapidly adapted into clinical practice^30^ in a vast number of eye diseases, such as diabetic retinopathy^6,31–33^ and age-related macular degeneration^34,35^; further, it was also implemented for imaging in neurodegenerative diseases^36^. So far, in most of these studies, a common 2D depiction of OCTA data has been used as a so-called en face representation^5,30^ which served as a trade-off between the amount of data and the capabilities of data representation.

As retinal vessels do not grow in two dimensions, the ordinary 2D OCTA display method is evidently only able to show limited areas of the retinal vasculature. Therefore, the scope of this study was to extend the current OCTA method with a novel approach into the 3D realm and to measure the naturally occurring variations of retinal vessel surface areas in healthy subjects^37–39^. This is of particular interest because the vascular surface in the retina represents the most important diffusion barrier and participates in the exchange of small molecules such as oxygen and glucose from the blood to the surrounding tissue. Thus, volume-rendered vessel surface data can be useful for example in diabetic retinopathy in which a decrease of pericytes is associated with a loss of the capillary network and therefore inevitably also from the vessel surface contributing to the end-stage and blindness^40^.

Furthermore, although normative en face OCTA data have been reported^18,19^, this is the first reference report on volume-rendered OCTA vessel surface area values to date: the mean overall vessel surface area was 54.43 mm^2^ for a retinal optical specimen measuring approximately 2.25 mm^3^. For the participants, the width of range from minimum to maximum varies up to 56% for males and up to 38% for females. This rather wide bandwidth is remarkably large, so it will be important for future studies to include a sufficiently large number of eyes if retinal vessels are to be compared.

One of the main findings of this study was that the vessel surface area was strongly correlated with the axial length of the eye (p = 0.049 for the right eye, p = 0.009 for the left eye). This result is in agreement with those of other reports^24,41,42^ using en face OCTA data.

The influence of sex and age on retinal OCTA perfusion has been a controversial topic as some studies have described a correlation, while others have failed to demonstrate an influence^19,27,43^. Possible reasons for this are the relatively small number of subjects in previous studies, different segmentation levels, and the simultaneous examination of both eyes; consequently, the scope for identifying correlations between the eyes could have been limited. However, in this study, the influence of common segmentation errors was avoided because the vessel volumes were rendered. This study has found a slight negative correlation between age and vessel surface area in right and left eyes. The negative correlation was significant in left eyes but not so in right eyes as revealed by tests for non-correlation (significance level 0.05). In order to investigate the joint influence of sex, age, SE, AL, and VA on the OCTA vessel surface area a GLM analysis was performed. Interestingly, the GLM analysis found a strong influence of AL in both eyes but it didn’t detect a significant influence of age on vessel surface area in neither left nor right eyes (significance level 0.05). This finding highlights the importance of considering more variables than just age and sex when studying OCTA perfusion. In this study, the slight negative correlation between age and vessel surface area turned out to be non-significant when including more explanatory variables in the model.

This study has a few limitations that should be noted. A key shortcoming of OCTA is that the image of blood flow does not correspond to the actual blood vessel lumen but a convolution of the flow as influenced by the OCT device and software used for flow detection^6,44^. Thus, OCTA signal saturates at a very low flow level and the imaged vessel diameter may be somewhat larger than the actual vessel diameter. This will make it difficult to interpret findings across different devices because every device uses different algorithms. Another limitation was that the OCTA data were exported as raw files and therefore projection artifact removal^44,45^ was not provided by the manufacturer’s software. Therefore, the measured values have to be considered with care. Nevertheless, the patient group was a fairly representative group with a healthy retina and no potential pathological inclusions that could trigger additional artifacts.

One issue in determining the true size of an object from OCTA measurements is the ocular magnification effect which states that the true size of an object in OCTA depends on the magnification factor of the eye^46^. This magnification factor is generally unknown even though there are methods to estimate it from axial eye length^47,48^. Regarding this study, it was decided not to apply any correction to the raw OCTA data since there are no published international standards. However, in Supplementary raw data S1 and S2 we provide, along with the OCTA retinal vessel surface area based on raw OCTA data, a possible type of correction of the surface area based on Littmann’s method^47^ and Bennett’s formula^48^. If there were standards for the correction of the ocular magnification effect, the correction of the values could be carried out quite easily. Nevertheless, the discussion remains yet unresolved whether these formulas are also applicable to a 3D environment. The advantage of the current data is that they are raw data without any modifications, which makes their further application more generalizable. The measured values are arguably valuable, but will only be of benefit when applied to retinal pathologies, which will be addressed in future studies. As already known with regard to conventional 2D en face OCTA, the data quality and OCTA artefacts^41^ will probably also influence the values obtained with volume rendering. This was counteracted by only including measurements with sufficient signal quality and a relatively high number of subjects.

A limit could be that currently no reproducibility test has been performed. Nevertheless, for the reproducibility of the described OCTA method, a mean intraclass correlation coefficient (ICC) of 0.845 or a mean ICC of 0.999, and an intraclass coefficient of variation of 0.07 or 0.0006, was reported earlier^39^ so that it is comparable to standard en face OCTA methods^49,50^.

Moreover, a limitation could be that no visual field measurements were recorded. Such a laborious procedure is unusual in routine clinical examinations where the data were collected for this study.

In summary, this study showed that it is possible to measure the vessel surface area using an expanded volume-rendered OCTA realm. This is important because the vessel surface area is the essential diffusion barrier for the exchange of metabolites and oxygen in the retina. It must be considered that the vessel surface area can be influenced by the AL of the eye and by a remarkably wide bandwidth within the healthy individuals.

## Methods

### Subjects

The study was conducted in accordance with the tenets of the Declaration of Helsinki. All the participants were seen at the Institut Clínic d’Oftalmologia, Hospital Clínic de Barcelona, Barcelona, Spain. OCTA images and relevant ocular and systemic clinical data were recorded prospectively during a 24-month period, as part of a larger prospective OCTA trial (ClinicalTrials.gov, trial number NCT03422965). All data were captured between 4 and 6 pm. This project was approved by the Institutional Review Board of the Hospital Clinic of Barcelona (HCB/2016/0216), and written informed consent was obtained from each subject. Each participant underwent a comprehensive ophthalmological examination, as described elsewhere^51,52^. The relevant ocular clinical data that were collected included best-corrected visual acuity (BCVA), SE, slit-lamp biomicroscopy results, intraocular pressure measurements, retinal fundus examination results, and AL (IOL Master, Carl Zeiss Meditec, Dublin, CA). The collected systemic clinical data included age, sex, smoking status, systolic and diastolic blood pressure, height, weight, and body mass index (BMI). A comprehensive battery of OCT and OCTA images was captured using a Cirrus 5000 OCT device (Carl Zeiss Meditec, Dublin, CA). The current device has only a limited function for the removal of projection artifacts which is only possible in the overlays "Deep", "Avascular", "Choriocapillaris", "Choroid" and does not allow this modality for the export of raw data.

The inclusion criteria were as follows: Caucasian origin, age ≥ 18 years, no history of any eye disease or neurodegenerative disease, the ability to fixate steadily, and clear optical media in the examined eye.

The exclusion criteria were refusal to provide written informed consent; the presence of diabetes, coronary heart diseases, or peripheral vascular diseases; amblyopia; high myopia above minus 6 diopters; and the presence of epiretinal gliosis or media opacities, including cataract.

### OCTA imaging procedure

A single OCTA volume scan of the central macula was performed per eye using a Spectral-Domain Cirrus® HD-OCT system (Carl Zeiss Meditec, Dublin, USA). This resulted in a one-volume OCTA scan, with dimensions of 3 mm × 3 mm × 2 mm, comprising 245 × 245 × 1024 pixels. Only scans with at least seven out of 10 OCT signal intensities, as displayed on the device, were selected.

### Image processing and total vessel surface area

**Figure 2:**
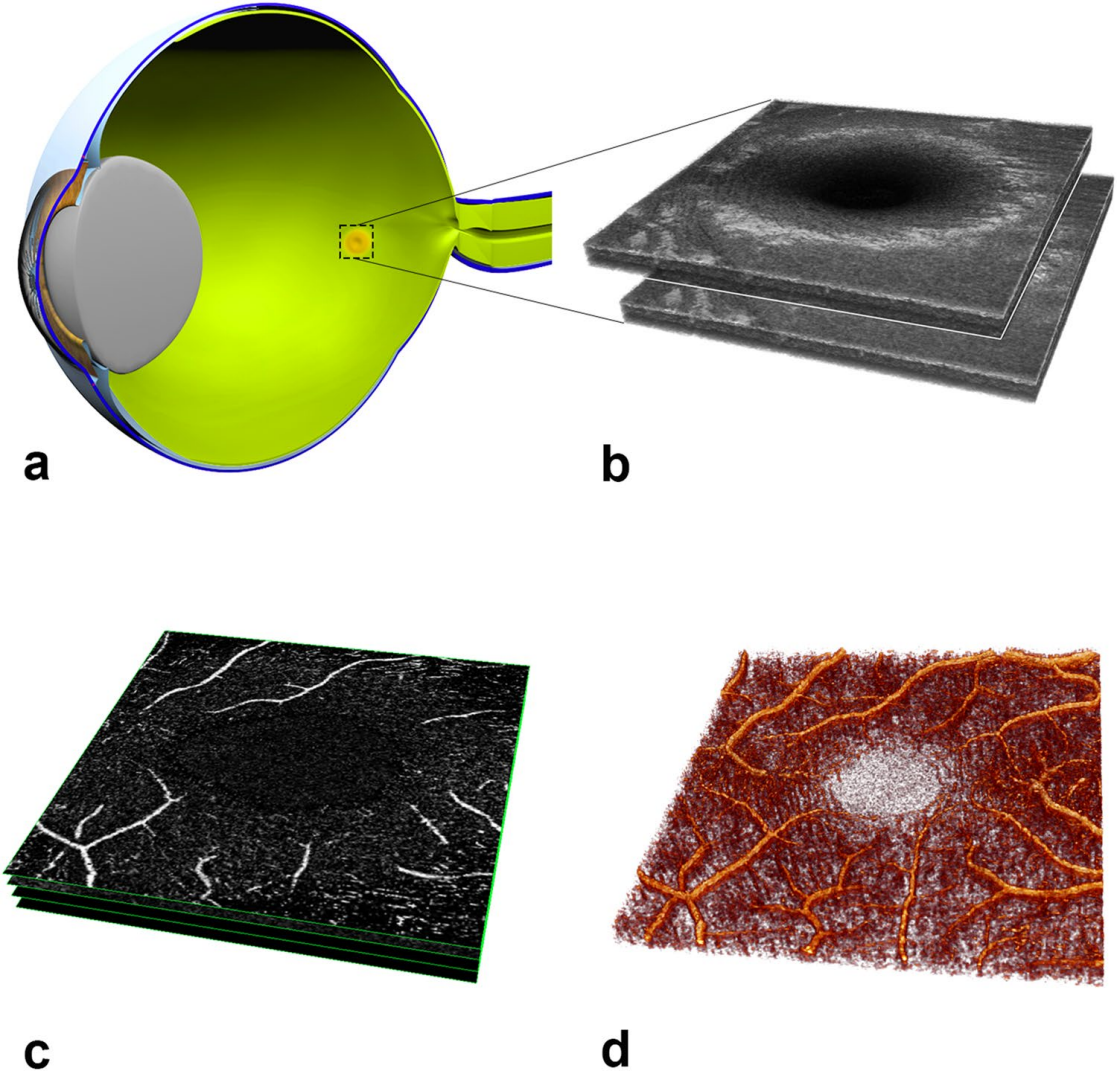
Presentation of image processing in three-dimensional optical coherence tomography angiography. (**a**) Schematic representation of an eye that has been cut open in order to provide a better explanation. In the posterior region, a brownish area is depicted, which corresponds to the location of the sharpest vision, the macula. Cross-sectional images of the macula in the area of the rectangle were captured from the same location. (**b**) These images correspond to structural OCT data that can be rendered into a volume representation. By repeatedly applying the same volume (here, two such volumes are shown as an example), the change in the OCT signal compared to the static tissue signal, which does not change much, can be interpreted as representing the blood flow. (**c**) Based on this, a common representation of the blood flow within the vessels can be shown via an en face image display method. Four such cross-sectional en face images are depicted here (each highlighted in green). However, as a trade-off between the amount of data and the processing, the intervening vessel parts are lost. (**d**) A three-dimensional rendering of the same data, showing the entire course of the vessels and their interrelationship. The rounded and vascular-free area in the center corresponds to the foveolar avascular zone (FAZ), in which the photoreceptors can interact as directly as possible with light.

OCTA data were exported from the OCT device as proprietary raw data in the .img image format using the software provided by the device manufacturer. Using the Zeiss raw data did not allow for the use of the direct artifact removal feature provided as part of the device manufacturer’s software. The images were automatically converted to one en face .bmp sequence per eye, resulting in an optical specimen of 3 × 3 × 2 mm per eye (Fig. 2). Image post-processing included the application of an automatic script written in MATLAB R2017a (MathWorks Inc., Natick, USA), through which the retinal flow signal was separated and made suitable for the measurement of the total 3D vessel surface area; details regarding this method, including its reproducibility, were previously reported on^53^. Briefly summarized:

The first step was to increase the contrast with the contrast limited adaptive histogram equalization (CLAHE). Afterwards, the images were processed with image sharpening using unsharp masking using built-in Matlab functions. The representation of the vessel signals was enhanced by using "vesselness" filters in order to be able to calculate the "vesselness" per pixel afterwards. The final step involved the application of a hysteresis threshold so that binary maps of the vessels were created from the preprocessed data. Finally, MATLAB provided the possibility to measure the entire vessel surface based on the voxel values.

The individual MATLAB parameters were as follows: CLAHE enhancement limit 1, number of bins 5, alpha distribution 0, vesselness filter minimum scale 10, maximum scale 15, lower threshold 40, upper threshold 45, and minimum size 30.

### Summary statistics and data plots

The following summary statistics were calculated across all eyes for the independent variables, age, SE, AL, VA, and OCTA retinal vessel surface area: mean, standard deviation, minimum, 1st quartile, 2nd quartile, 3rd quartile, and maximum values. In addition, summary statistics were calculated separately for male right eyes, female right eyes, male left eyes, and female left eyes. For visualization, univariate box plots and scatter plots were generated by plotting one independent variable at a time versus the OCTA retinal vessel surface area. Plots were generated separately for the left and right eyes. Summary statistics were calculated in Python v3.8^54^ with pandas v1.1^55^ and boxplots were generated in Python with Matplotlib v3.3^56^.

### Correlation analysis

Pearson correlation coefficients were calculated (1) between OCTA retinal vessel surface area and age and (2) between OCTA vessel surface area and AL. Furthermore, tests for non-correlation were performed based on the Pearson correlation coefficients. Right and left eyes were investigated separately. Calculations were done in Python v3.8 with scipy v1.6^57^.

### GLM analysis

A GLM analysis was performed on the left and right eyes separately in order to investigate the main effects of the independent variables, sex, age, SL, AL, and VA, and all the possible two-way interactions with the dependent variable, OCTA retinal vessel surface area. Sex was treated as a discrete variable, and age, SL, AL, and VA, as continuous variables. Before the analysis, two outliers were removed in the case of the left eye. The GLM analysis was performed in R v3.6^58^ with car v3.0^59^ using the type II sum of squares estimation method. The interaction terms that were found with the type II sum of squares analysis were also found with the type III sum of squares analysis (with the same significance codes), which is more reliable for identifying interaction effects (type III sum of squares results are not shown). GLM analyses were performed to identify the link function and Gaussian noise.

### Right vs. left eyes

A two-sided paired *t*-test was performed to investigate whether the left and right eyes differed in terms of the OCTA retinal vessel surface area. For this purpose, data from 94 subjects, whose left and right eye data were recorded, were used. The *t*-test was performed in Python v3.8 with scipy v1.6^57^.

## Supplementary Information

Supplementary Information 1.

Supplementary Information 2.

## Data Availability

The datasets generated and analyzed during the current study are available in Supplementary raw data S1 for the right eye and Supplementary raw data S2 for the left eye.
